# Impact of Ventilatory Modes on the Breathing Variability in Mechanically Ventilated Infants: A Commentary

**DOI:** 10.3389/fped.2014.00147

**Published:** 2015-01-14

**Authors:** Maroun J. Mhanna

**Affiliations:** ^1^Department of Pediatrics, Metro Health Medical Center, Case Western Reserve University, Cleveland, OH, USA

**Keywords:** pediatric intensive care, mechanical ventilation, neurally adjusted ventilatory support, diaphragm, children

Neurally adjusted ventilator assist (NAVA) ventilation is a mode of ventilation that is triggered and cycled by a signal from the electrical activity of the diaphragm (EAdi) to provide a positive pressure breath. The EAdi signal is obtained from an esophageal catheter that is positioned at the level of the diaphragm. The rational of this mode of ventilation is to improve patient–ventilator interaction by matching ventilator support to patient’s demand and therefore avoiding hyperventilation and air trapping, and impairment of cardiac output (1). Several studies have shown that NAVA is better tolerated than other modes of mechanical ventilation in infants and children. For instance, premature infants require a lower peak inspiratory pressure (PiP) and a lower fraction of inspired oxygen (FiO2) and they have a better blood gas regulation at a lower respiratory rate when ventilated with NAVA instead of pressure control (PC) (2). Furthermore, premature infants have a reduction in their respiratory muscle load and a lower PiP when ventilated with NAVA instead of synchronized intermittent mandatory ventilation (SIMV) with pressure support (PS) (3). Also, critically ill children are more comfortable when ventilated with NAVA instead of PS. They have a better synchronization with the ventilator, a reduction in their ventilatory drive, and an increase in breath to breath variability while on NAVA (4).

Baudin and colleagues attempted to compare the impact of different modes of ventilation on the respiratory drive patterns in infants. The authors used the non-rhythmic to rhythmic (NRR) index as a method to assess the EAdi variability pattern and analyze respiratory variability during NAVA, PC, and PS ventilation. They conducted a retrospective study comparing the EAdi variability pattern of 10 ventilated infants to a control group of 11 non-ventilated spontaneously breathing infants. Infants in the control group were not intubated at the time of their study, but they had an esophageal catheter for evaluation of their EAdi pattern. The authors’ main findings were that mechanical ventilation impacts the breathing variability and NAVA produces an EAdi pattern that resembles most of that seen in non-intubated mechanically ventilated infants (5).

While the authors are applauded for taking on the challenge to study the effect of NAVA on the respiratory drive pattern in critically ill children, their study suffers from a major limitation. Their design was retrospective in nature. Their *post hoc* analysis of two previous studies weakens their argument that EAdi variability on NAVA resembles the endogenous respiratory drive pattern of healthy children. For a stronger argument, the authors’ hypothesis should have been *a priori*. The study also suffers from a poor control group. Their control group was younger and smaller than the mechanically ventilated group. And since periodic breathing is more prominent in younger than older infants, the control group is more prone to periodic breathing than the ventilated group. Therefore, the presence of periodic breathing becomes a confounder. A prospective study using patients as their own controls will counteract potential confounders related to age, weight, or breathing patterns. For instance, a comparison of the same patients’ EAdi variability patterns before and after extubation from NAVA ventilation will address such confounding variables.

Above all, the most important question remains unanswered “does NAVA improve the long-term outcome of children with acute respiratory failure?” Future prospective studies are needed to determine if the short term beneficial effect of NAVA has any long-term effect on the duration of mechanical ventilation, need for sedation, intensive care unit length of stay, and mortality in critically ill children.

## Conflict of Interest Statement

The author declares that the research was conducted in the absence of any commercial or financial relationships that could be construed as a potential conflict of interest.
